# Challenges and Enablers of Deprescribing: A General Practitioner Perspective

**DOI:** 10.1371/journal.pone.0151066

**Published:** 2016-04-19

**Authors:** Nagham J. Ailabouni, Prasad S. Nishtala, Dee Mangin, June M. Tordoff

**Affiliations:** 1 School of Pharmacy, University of Otago, Dunedin, New Zealand; 2 David Braley Chair in Family Medicine, McMaster University, Hamilton, Canada and University of Otago, Christchurch, New Zealand; Royal College of Surgeons, IRELAND

## Abstract

**Aims:**

Deprescribing is the process of reducing or discontinuing medicines that are unnecessary or deemed to be harmful. We aimed to investigate general practitioner (GP) perceived challenges to deprescribing in residential care and the possible enablers that support GPs to implement deprescribing.

**Methods:**

A qualitative study was undertaken using semi-structured, face-to-face interviews from two cities in New Zealand and a purpose-developed pilot-tested interview schedule. Interviews were recorded with permission and transcribed verbatim. Transcripts were read and re-read and themes were identified with iterative building of a coding list until all data was accounted for. Interviews continued until saturation of ideas occurred. Analysis was carried out with the assistance of a Theoretical Domains Framework (TDF) and constant comparison techniques. Several themes were identified. Challenges and enablers of deprescribing were determined based on participants’ answers.

**Results:**

Ten GPs agreed to participate. Four themes were identified to define the issues around prescribing for older people, from the GPs’ perspectives. Theme 1, the ‘recognition of the problem’, discusses the difficulties involved with prescribing for older people. Theme 2 outlines the identified behaviour change factors relevant to the problem. Deprescribing challenges were drawn from these factors and summarised in Theme 3 under three major headings; ‘prescribing factors’, ‘social influences’ and ‘policy and processes’. Deprescribing enablers, based on the opinions and professional experience of GPs, were retrieved and summarised in Theme 4.

**Conclusion:**

The process of deprescribing is laced with many challenges for GPs. The uncertainty of research evidence in older people and social factors such as specialists’ and nurses’ influences were among the major challenges identified. Deprescribing enablers encompassed support for GPs’ awareness and knowledge, improvement of communication between multiple prescribers, adequate reimbursement and pharmacists being involved in the multidisciplinary team.

## Introduction

Deprescribing describes the process of reducing or discontinuing medicines that are un-necessary or deemed harmful [1, 2]. Deprescribing is a proposed antidote to the harms of polypharmacy and is associated with numerous health benefits including improvement in cognition [3], a reduction in falls [4], a decrease in fractures [5], better medication adherence [6] and improvement in quality of life [3].

Deprescribing is an inherent component of good prescribing practice [7], but is rarely implemented in routine clinical care, and physician practice or views on deprescribing vary greatly [8, 9]. It is important to recognise that deprescribing involves more than just identifying inappropriate medicine use (IMU) [6]. It involves reviewing all medicines prescribed for the patient, identifying those medicines which are deemed un-necessary and potentially harmful, deciding which medicines can be stopped and considering the order in which medicines are to be stopped, tapered or reduced, with adequate monitoring and follow up. A deprescribing medication management plan is a final step of this process, and should be centered on the patient’s preferences where possible.

The prevalence of single-disease guidelines for initiating treatments is a significant driver to polypharmacy and is a hindrance to deprescribing [10]. As of late, drug-specific deprescribing guidelines have been a research focus as a potential solution. A recent study by Farrell *et al*. used Delphi processes to prioritize medications where guidelines for deprescribing would be of benefit to clinicians [9]. The feasibility of deprescribing proton pump inhibitors (PPI) was examined by Reeve *et al*. using an example of a drug-specific PPI deprescribing guideline [11].

The lack of availability of such deprescribing guidelines may be one of the barriers to GPs implementing deprescribing [12]. On the other hand, a possible enabler to deprescribing may include, physicians and pharmacists working together to carry out comprehensive medicine reviews aimed at reducing polypharmacy and inappropriate medication use (IMU) in older people [13–16].

However, to date, very limited information exists on such barriers or challenges and possible enablers for deprescribing. In addition, limited information on GPs’ opinions regarding working closely with pharmacists in a multidisciplinary team, exists. Therefore, this study aimed to ascertain these challenges and enablers, by examining the views of GPs about deprescribing for older people in a residential care setting. The specific aims of this research are to:

1. Explore challenges faced by GPs to deprescribing in the residential care setting.
2. Explore enablers that facilitate deprescribing in the residential care setting.
3. Explore views on deprescribing guidelines and a possible role for pharmacists in the process
4. Use the Theoretical Domains Framework (TDF), consisting of theoretical constructs, to help understand the possible barriers and enablers of deprescribing in a residential care setting.

## Methods

A qualitative design was used for exploring the views of general practitioners prescribing for older people in a residential care setting. An interview schedule (Table 1) comprised of six questions was developed that aimed to explore GPs’ challenges when prescribing for older people in a residential care setting. The scope of the interview schedule also covered the factors taken into consideration by physicians when prescribing, opinions on deprescribing guidelines. The study’s interview schedule specifically explored GPs’ opinions on the possible role of pharmacists in the multidisciplinary team to conduct a deprescribing medicine review. The interview also included a hypothetical patient profile adapted with permission from a study published by Schuling *et al*. [12]. Adaptations included providing additional clinical information such as renal function and body weight. In addition, the physician who initially prescribed the medicine was identified. The interview schedule was piloted on three GPs and modified in light of their comments. The present study reports on the qualitative data collected from the six primary interview questions. Data and discussions arising from the hypothetical patient profile will be reported separately.

**Table 1:** Interview questions and prompts.

How do you feel about prescribing for older people living in residential care? Prompts:
*Challenges of prescribing in this setting*
*Ease or difficulty of reviewing older people’s medicine profiles*
*Clarity of residents’ clinical notes and medicine charts*
*Communication*, *including clarity of documentation*, *at transfer of points of medical care and (e*.*g*.: *hospital discharge)*
When prescribing medicines for these patients, what factors do you think are important to consider? Prompts:
*Patient factors (e*.*g*.: *quality of life*, *benefit gained versus risk caused)*
*Physician factors (e*.*g*.: *prescribing habits*, *personal preferences*, *past experience)*
*Other factors (e*.*g*.: *residential care staff*, *other prescribers*, *patient/relatives’ wishes)*
How do you approach reducing or stopping medicines in older people living in rest homes? Prompts:
*Do they endorse this idea*? *Do they have any concerns*? *(patients’ or relatives’ views)*
*Do they find stopping medicines challenging*? *Why*?
*How frequently do they tend to stop medicine(s)*?
*What factors do they take into account when making those decisions*?
*Do deprescribing decisions occur at the resident’s regular clinical review multi-disciplinary meetings or at another time*?
If there were a guideline designed to assist prescribers in making decisions around deprescribing in older people, would you consider this to be useful for your clinical practice?
*What type of guidance would they find useful*?
*Would they find deprescribing guidelines helpful or burdensome*?
*Would guidelines make it easier or more efficient to review residents’ medicine lists*?
What do you think of a clinically trained pharmacist or a prescribing pharmacist being involved in the process of reviewing residents’ medicines? Could pharmacists make clinical recommendations for the GPs’ consideration and discuss them with the multi-disciplinary team during the regular clinical reviews?
*Do they think a pharmacist’s involvement in this process would reduce the GPs’ workload*?
*What are the possible challenges in involving a pharmacist in this process*?
Is there anything you think you would like to help you with this process of reducing/stopping medicines (i.e. deprescribing)? Is there anything that could make this process easier?

Ethics approval was granted by the University of Otago Research Committee (Ethics Approval Number: 14/038). This was not a quantitative study requiring an epidemiologically representative sample. As information on doctor characteristics are not publicly available, randomization was a convenient way to obtain our sample and start to investigate purposively to ensure it had a range of characteristics. Using information available from New Zealand district health board (DHB) websites, a list of all medical centres in the two cities was complied. A random number generator programme was used to produce 10 numbers. The ten medical centres that correlated to these numbers were approached for recruitment. NA used the medical centres’ websites to find the names of the doctors who currently worked in each medical centre. Personalized invitations were accordingly sent out to each potential participant. GPs that provided clinical care for ≥ 10 residents living in residential care were invited to respond. After a month, invitation letters and information sheets were sent out to a second randomised list of medical practices in the same towns/cities. GPs who agreed to participate provided written consent. Signed consent forms were sent back in pre-paid reply envelopes.

We have used the consolidated criteria for reporting qualitative research (COREQ-32) to report important aspects of the research team, study methods, context of the study, findings, analysis and interpretations (Table 2).

**Table 2:** COREQ-32 checklist.

Domain 1: Research team and reflexivity	
*Personal characteristics*	
Interviewer	Nagham Ailabouni (NA)
Credentials	PhD candidate
Occupation	Pharmacist
Gender	Female
Experience and training	Carried out research in residential care; worked in hospital and community pharmacy
*Relationship with participants*	
Relationship established	GPs were selected randomly; no relationship existed prior to interview
Participant knowledge of the interviewer	Participants did not know the interviewer prior to the interview.
Interviewer characteristics	No characteristics were reported
Domain 2: Study design	
*Theoretical Framework*	
Methodological orientation and theory	Content analysis. Findings, such as themes, were drawn from data collected during interviews.
*Participant selection*	
Sampling	Random sampling of medical centres
Method of approach	Mail
Sample size	10
Non-participation	No participants dropped out. 30 GPs did not reply to participate.
*Setting*	
Setting of data collection	Medical clinic
Presence of non-participants	No
Description of sample	Number of years’ experience prescribing in residential care: 2–32 years; Ethnicity: 8 NZ European, 1 European and 1 Asian participant; Gender: Male (7); Female (3)
*Data collection*	
Interview guide	The questions were written by the authors, and prompts were given during the interviews if needed (Table 1). Interview schedule was pilot tested on three GPs.
Repeat interviews	No
Audio/visual recording	Audio recording was used
Field notes	NA made field notes during interviews when necessary
Duration	15–25 minutes
Data saturation	Yes. Data saturation was reached when the major and minor themes were repeated in interviews. Coding was independently checked by a second investigator (JT).
Transcripts returned	No
Domain 3: Analysis and findings	
Number of data coders	One (NA)
Descriptions of the coding tree	The Theoretical Domains Framework (TDF) was used as a basis for the coding tree
Derivation of themes	Themes were derived from data collected
Software	nVivo 10
Participant checking	It was agreed with participants, that findings will be shared with them upon publication
*Reporting*	
Quotations presented	Yes
Data and findings consistent	Yes
Clarity of major themes	Major themes resulting from the interviews are outlined in this publication.
Clarity of minor themes	The patient profile is considered a minor theme in this study. This will be reported in a follow up study. Participant responses were analysed in depth. Findings from this were discussed and compared with evidence based research available in older people and other similar studies carried out.

The interviews conducted were in depth semi-structured face-to-face interviews, using the interview guide in Table 1. This interview method for data collection was chosen over a focus group method, as it was thought to be more time-efficient for the GPs, would avoid any particular views being dominant in a meeting, and would allow them to express an opinion without fear of being judged by their colleagues. Interviews were recorded with permission and transcribed verbatim. Transcripts were read and re-read and themes identified, with iterative building of a coding list until all data was accounted for. Interviews continued until saturation of ideas occurred.

Analysis was carried out using constant comparison techniques, and the nVivo 10 software. A Theoretical Domains Framework (TDF) developed by Michie *et al*. [17], was used to assist with the analysis (Table 3). The Michie TDF was considered most appropriate as it was specifically developed for the purposes of implementational research. It was developed by a robust consensus approach, is commonly used in health research. This framework consists of 12 domains or theoretical constructs that help determine the barriers and facilitators of implementing any change in behaviour or practice. In this study, the practice or behaviour that is being examined is deprescribing. Several sub-domains or specific beliefs were drawn up for each domain which relate to analysing the behaviour of the GPs in relation to deprescribing. Interview quotes to support each domain are listed, as appropriate in Table 3. As the TDF does not include domains specific to the challenges/enablers of deprescribing, a simple thematic analysis was applied to Table 3 to draw themes that related to the challenges of deprescribing (Theme 3) and deprescribing enablers (Theme 4). Coding was initially conducted by NA, and independently checked by JT. Coding was discussed, and any possible discrepancies were resolved. Interpretations were checked by JT, PN and DM.

**Table 3:** Behaviour change factors related to deprescribing.

Domain	Sub-domain/specific belief	Sample Quotes
Knowledge	GPs’ knowledge about deprescribing.	*“Why don’t we deprescribe*? *Is that a knowledge issue*? *Possibly*. *Its very complicated*. *Do we know all the interactions and side effects of all these drugs*? *Of course we don’t*.*” GP4*
	Uncertainty about the relevance of evidence based guidelines to older people with multimorbidity.	*“But you don’t have guidelines a lot in the elderly*, *do you*? *That’s the hardest thing*.*” GP10*
	Lack of guidelines relevant to prescribing in older people with multimorbidity.	*“Often research will say if you’re above for example*, *80 (years of age) you’re excluded*.*” GP9*
Skills	Difficulty determining medicines to deprescribe, and appropriate timing of deprescribing.	*“And also there’s the challenges of when do you stop preventative stuff*, *when do you stop your statins and aspirin*?*” GP3*
Professional role and identity	Trying to fulfil professional duties, despite struggles.	*“By the time you’ve crossed some off and put some new ones on*, *you have to re-do the chart and that just takes time*. *I have to charge for that because it takes the time*, *I don’t know if there’s any better other way*.*” GP10*
		*“I don’t have that much time… for those kind of moral routine stuff*, *because I already have a lot of acute stuff I got to deal with on the days that I go there*.*” GP9*
Motivation and goals	Competing factors (time, rest home policies, other prescribers etc.) decrease motivation to deprescribe.	*“I don’t really know what the evidence is*, *but I suspect that physicians don’t deprescribe very much*.*” GP4*
	GPs’ motivation to deprescribe.	*“Since I’ve started to look at that more globally*, *the number of medicines I’m prescribing on average for patients in rest homes is about 50% of what I was prescribing a year ago and they aren’t falling off their perch in greater numbers*. *Patients like it (being prescribed less medicines)*. *They say oh*, *a whole big meal of pills*, *and you know*, *people are generally better*. *People wake up*, *they’re less nauseated*, *they have fewer falls*, *all those sorts of things*, *yeah*. *GP5*
Memory, attention and decision processes	Attention and effort needed to deprescribe.	*“It’s because*, *obviously you’ve got a clinical responsibility*. *Stopping a medicine is in a way no less a therapeutic position than starting a medicine*. *So you’ve still got to then consider the down flow effects of that on the patient*, *so you need a management plan” GP4*
Environmental constraints	Access to clinical notes.	*“You rush at lunchtime or you rush before work*, *so you’re often fitting it (rest home prescribing) in around other things*.*” GP2*
	Multiple competing demands of professional role	*“Well we’ve got the notes back at the surgery*, *but they’re not linking up with the rest home*. *Reality would be to move onto some kind of computerised system” GP3*
	Lack of decision-support systems.	*“Rest home prescribing doesn’t fit well with my schedule*. *It is a bit of a juggling act*, *so I don’t personally like it*. *If you go to rest homes*, *some of them are like 15 minutes each side*, *so doesn’t really fit with me*, *and even when you say ok I’ll go about 12 or something quickly*, *they’re eating*. *They’re in their lunch*, *so you just end up waiting*. *Other thing is that you see them at the end of the day*, *which is again you know*, *six or something*, *and then again if you need something*, *or medication*, *the pharmacies are closed up*.*” GP8*
	Accessibility of the resident or patients	
	Time constraints	
Social influences	Lack of adequate reimbursement. Communication at points of health care transfer	*“I do think sometimes*, *you wonder who are we treating*? *Are we treating the nursing staff who can’t face somebody calling out at night*, *or are we treating actual patient who may be very well*, *happen to be calling out once or twice a night at one o’clock*, *but then fall back to sleep*, *you know*?*” GP 9*
	Influence of nurses’ suggestions	*“If it is an important decision*, *then I’ll involve the family*. *But with some decisions*, *the family don’t need to know everything*.*” GP6*
	Patient’s ability to communicate.	*“Some patients are in hospital level care and the majority can’t even speak*.*” GP9*
	Patient beliefs, ideas, concerns or preferences	*“The reverse of hospitals putting them on medications*, *is that hospitals stop medications sometimes*, *and a week later they start them on all of the medicines again*, *so that sort of communication needs to be taken into consideration*.*” GP1*
	Involving family members or relatives	*“I think it’s also because the communication could be far better between the hospital and us” GP9*
		*“The challenges are stopping hospital physicians giving unnecessary medicines*.*” GP5*
		*“Once they go into a rest home*, *you haven’t got time to visit or a visit might not be relevant*, *so we’re relying on the nursing staff to feedback information to us*, *and often that’s not brilliant*, *hence we’ve lost that kind of one to one with the patient*, *which makes it much harder to decide how to prescribe*, *because you’re being biased by other people*.*” GP3*
Emotion	Fear of potential negative outcomes from Deprescribing.	*“The other reason people don’t like stopping medications*, *is uh*, *coincidentally people would die when you stop things*.*”GP3*
	Reluctance to change medicines prescribed by a specialist.	*“So if they’ve seen a cardiologist and were put on a statin*, *you feel very nervous about stopping it for example*. *There’s no doubt that you know a specialist assessment*, *commenced on a specific drug*, *you know it does make you reluctant to the change*. *To change things*, *yeah*, *it does have that*, *it influences you*.*” GP7*
	Lack of acceptance of GP decisions from other health professionals.	*“Everyone is having a go at us*. *Every speciality across the land will tell us that we should do things better*. *And pharmacists will tell us we should do things better*, *which is like; you need to come on our side of the fence and be a general practitioner*.*” GP4*
Behavioural regulation	Recognising the need to try and discuss therapy options with the patient	*“I’d do some discussion with the patient if possible*. *And I would do it on the basis of trying to work out the risks versus the benefits*.*”*
	Awareness of hindrances that prevent behavioural change	*“I suspect (deprescribing) guidelines and all the rest of it don’t change our behaviours*, *because you’re talking about behavioural change*.*” GP4*
		*“I don’t tend to look at those (deprescribing) guidelines*, *that’s the problem*. *There has been some*, *and I’ve got them to read my one day pile*. *But you never sort of quite get round to it*.*” GP10*
Nature of behaviour	Variance in frequency of implementing deprescribing	*“I don't have that much time for those kind of moral routine stuff*, *cause there’s already a lot of acute stuff you have to deal with on the days that I go to the rest home*.*” GP9*

## Results and Discussion

In total, forty invitations were sent to individual GPs and ten consented to take part in the study, giving a participation rate of 25%. Eight GPs were New Zealand European, one GP was Asian and one GP was Dutch. Participants had been prescribing for older people in residential care for 2–32 years. Four main themes were identified in the analysis of data from respondents which collectively describe prescribing in the residential care setting, from a GP perspective. Data also illustrate the various challenges involved in implementing deprescribing and possible deprescribing enablers.

### Theme 1: Recognition of the problem

In order to provide solutions to a problem, it is important to firstly define, recognise and acknowledge the extent of the problem. While several GPs stated that prescribing for older people in the residential care setting is no different to prescribing in general practice, the majority of them believed that prescribing is particularly challenging in residential care for a number of reasons.

*“Prescribing for older people in residential care*, *is no different than just general prescribing*, *but its harder*.*”*

GP10

*“Always a little nervous about prescribing for older people in rest homes*, *you know*, *because they often have multiple pathologies*.*”*

GP6

Multimorbidity is a common occurrence in old age, affecting over 50% of people in primary care [18]. As a result, patients are often prescribed multiple medicines. In addition, advanced age and frailty contribute to a reduced life-expectancy [12]. Therefore, physicians often have to balance a multitude of factors including the disease(s) the patient may have, the benefit-risk profile of medicines prescribed, the patients’ personal views and the opinions of other prescribers. This was identified as a key aspect of the perceived challenge in prescribing in rest home patients.

*“Firstly*, *there’s the patient themselves*, *and the fact that they are in a rest home*, *then there’s whatever disease you are treating*. *The point is*, *you know how the elderly are; they’ve got hundreds of things wrong with them*, *multiple pathologies and polypharmacy are the issues*.*”*

GP4

*“Basically*, *older people especially those in rest homes tend to have a lot of co-morbidities*, *so they are being prescribed things for a lot of conditions*. *They also have seen lots of specialists who have also prescribed things*, *and one of the challenges is knowing when you can take control and either stop or reduce doses*.*”*

GP1

Polypharmacy was highlighted as a major challenge for prescribing for older people by several GPs in this study. When GPs attempt to review prescribed medicines, they often queried the original indication these medicines were prescribed for. In addition, it was difficult for GPs to address polypharmacy as they are often trying to differentiate between medical conditions, or symptoms due to medicine side effects, and balance the potential for beneficial effects with the potential for side effects when making decisions around medication use. In another qualitative study examining GPs’ views by Schuling *et al*., this was also found to be a significant contributor to the problem [12]. Clinical decisions made by GPs about the appropriateness of medicines were sometimes based on very scarce clinical information.

*“How long have they been on it*?*” I have seen people on statins in their 80’s and 90’s for goodness sake*, *for non-STEMI’s*, *and you’re thinking*, *do they really need that*?*”*

GP10

*“Probably that’s a big challenge*. *Trying to keep everything appropriate*, *the right treatment for the right problem*, *but without overdoing it or overmedicating*. *Trying to get a balance between you know a good outcome and the person doing well*, *and the side effects from those medications*, *so side effects would be another issue in elderly*. *You can start a medication*, *and you’re getting to know other effects from that medication*, *so I guess that’s a doctor-induced problem*. *That’s the challenge when you’re prescribing for the elderly*.*”*

GP4

### Theme 2: Behavioural change factors

It was pertinent to examine and understand the behavioural change factors or drivers which contribute to the problem or enable implementation of solutions. The results are presented using the Michie TDF analysis domain template (Table 3).

### Theme 3: Deprescribing considerations and challenges

**Fig 1:**
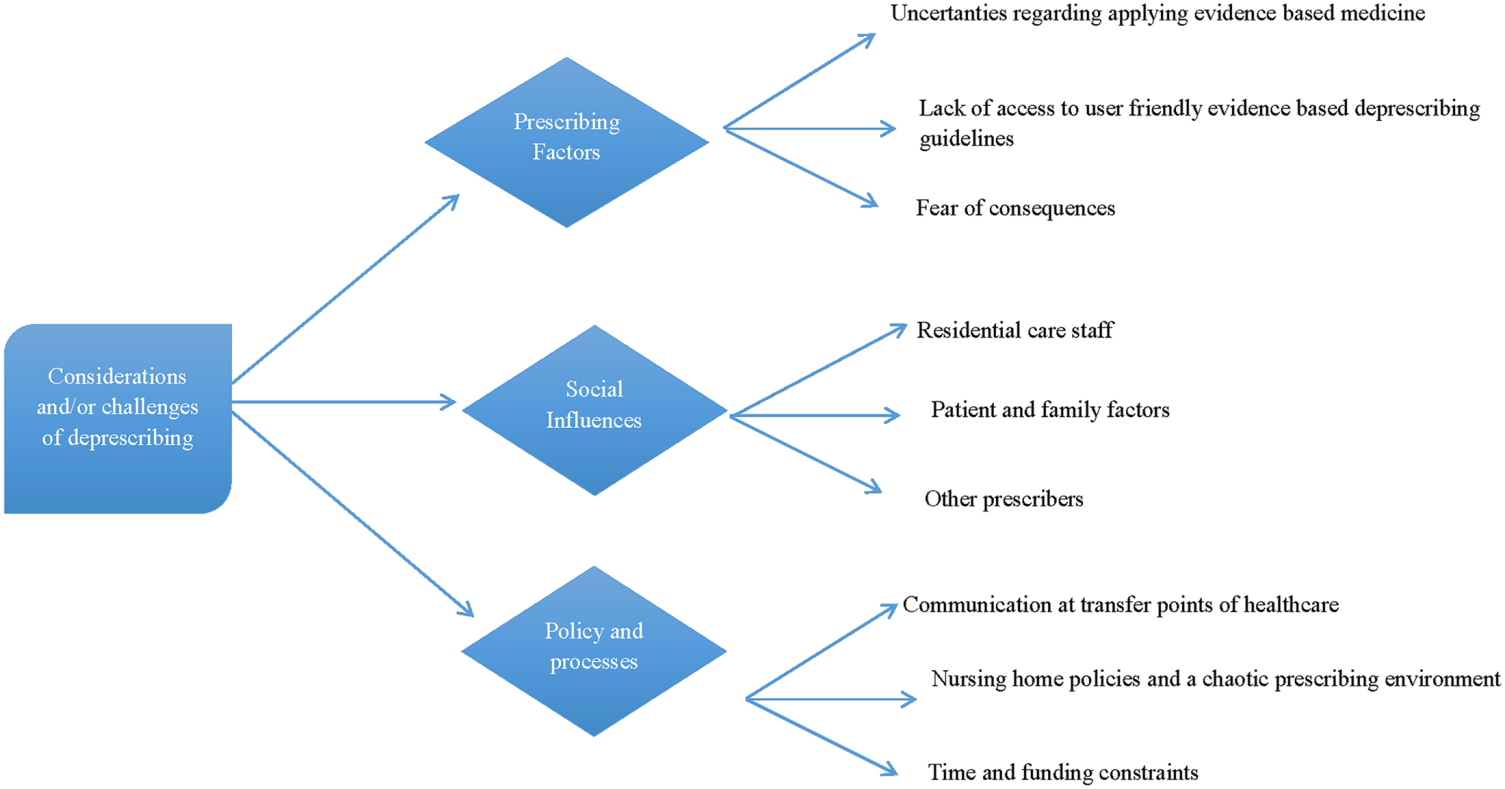
Deprescribing considerations and challenges.

Analyses of the behavioural change factors (Table 3) highlighted several deprescribing considerations and challenges (Fig 1). These were grouped under three main headings, prescribing factors, social influences and policy and processes.

#### A. Prescribing factors

Overall, GPs acknowledged that it is important to consider the quality of life, health status and life expectancy of their patients, prior to initiating a medicine [12].

“Once they go into the rest home, they’re obviously only going to live for another 5 years potentially, I don’t know what the actual figures are. So you look at it in that view point. You know, everything changes. You look at the risks and benefits of every single decision you’re making.” GP3

“Patient factors would be um, their life expectancy. That’s putting it bluntly, isn’t it? Their age, I think you know, if someone’s you know, you might consider treating a 72 year old different from a 92 year old, or a 102 year old, had a few of those.” GP 4

**i. Uncertainties regarding applying evidence based medicine:** GPs’ knowledge and ability to synergise and apply evidence based practice may be challenged when caring for older people with complex health problems [19]. GPs in this study acknowledged that they experienced a dilemma when applying research evidence to a patient with multimorbidity. This was the case particularly for preventive medicines such as aspirin and statins.

“There’s also a challenge of when do you stop preventive medicines such as statins and aspirin?” GP3

The dilemma of continuing or stopping preventive medicines also concerned GPs who reviewed a patient profile in our recent qualitative study. Our study found that GPs felt more confident about symptom management and adjusting the dose of symptomatic medicines, according to patient response [12]), rather than reviewing the appropriateness of continuing preventive medicines.

Lack of access to user friendly evidence based deprescribing guidelines: Few clinical trials have highlighted that nonadherence to disease-specific clinical guidelines may result in poor quality of patient care [20]. Paradoxically, there is growing evidence that older people with multiple conditions have poorer outcomes when treated according to disease-specific guidelines compared to other patients [10, 21]. In addition, the presence of multiple guidelines discourages physicians from applying these guidelines in clinical practice [22]. One GP in the present study expressed their frustration regarding the complexity of guidelines.

“I have seen these Best Practice Advisory Committee (bpac^nz^) articles, but I can’t remember what was in them, you know? Has it changed my prescribing? No it hasn’t, which I suspect applies to a lot of us. We get so many guidelines, there’s so many… that’s the other thing. You have all this stuff (different guidelines) coming at you and everybody’s saying you could do better, and well maybe we could, in this speciality, but actually, when you’re putting it altogether, maybe we’re not as bad as people like to make out because when you’re sitting there with a lot of stuff, I mean we basically see that balancing huge amounts of information.” GP4

“Well like you mentioned earlier, that Best Practice Advisory Committee (bpac^nz^) one they’ve written about it, and just making you more aware and giving the time so that I can actually read it, I think I’ve started it, but I haven’t read it. It’s in a pile, so makes you more aware and more confident of making the changes if and when needed.” GP10

Lack of easy-to-use guidelines or decision support is a particular challenge for GPs whilst implementing deprescribing and many physicians are forced to make decisions without much guidance.

“Often research will say if you’re above for example, 80 (years of age) you’re excluded.” GP9

“And that’s the hard thing, whether a little bit of omeprazole, which you’re meant to slowly reduce from 40, to 20, to 10, to stop, and when should that be done? It’s not easy, because I mean guidelines say that you should reduce, but it is often not indicated why people are on say, omeprazole. I find those sort of things hard.” GP10

In a synthesis of ten qualitative studies, physicians highlighted that guidelines focus on health outcomes that may be of little relevance to their patients [22]. This leads to physicians deviating from guideline-directed care. In addition, they mentioned the lack of available tools to help physicians quantify the benefits and harms of prescribed medicines, as this is often a dilemma whilst optimizing medicines in older people. As a result, physicians making a clinical decision, follow an approach that combines prioritizing the main problems most relevant to the patient, stratifying the risk of a disease outcome, modifying guideline-directed goals or interventions according to anticipated adverse effects the patient might experience and then making a clinical decision balancing potential benefits and harms [22, 23].

**ii. Fear of consequences:** Physicians have a genuine fear of patients experiencing deterioration in their health, shortly after they have stopped or reduced one or more of their medicines. They may also fear others (family or health care staff) having the misperception that coincidental adverse effects the patient may develop, are a direct outcome of the physician stopping medicines.

“Coincidentally, people would die when you stop medicines. Not because you killed them, but they happened to.” GP3

In addition, physicians fear that patients may perceive deprescribing as withdrawal of care before their end of life. These misconceptions prevent physicians from implementing deprescribing. For many GPs, ‘maintaining the status quo’ for multimorbid patients with polypharmacy who are stable, is seen as the most reasonable course of action to undertake.

“If they’re otherwise stable, and life’s going on, it’s the same as prescribing for anybody else really which doesn’t really help you very much. I don’t reduce or stop unless I think it’s needed. If you stop it, are you going to de-stablilise things? So, if the system is working, I have to say, although we look and we wonder, we tend to leave them on.” GP4

“It’s hard to give them the option sometimes, and you feel like you’re writing them off if you’re crossing off all this preventative sort of stuff.”

Even though this fear of consequences exists, some GPs commented that through their personal experience, patients have often improved once their medicines have been stopped.

“I had a patient today, a 90 year old, I reduced her metformin and she said: I’m feeling much better since you did that, you now? So you can often get away with you know, reducing down.” GP6

#### B. Social influences

**i. Patient and family factors:** Several GPs in our study felt the importance of delivering individualised patient-centered care to their patients. This is similar to the views of other GPs who felt the importance of considering the patient’s personal preference and patient empowerment when tailoring care [24]. To achieve this, taking into consideration individuals’ opinions or perceptions about their medicines is important. However, patients may not always be willing to stop or change medicines they have taken for a long time [12], despite the physician’s recommendations.

“Sometimes it is hard because they have been on it for donkey’s years, and they totally believe in it.” GP9

In addition, certain patient characteristics, such as loss of cognition and memory, can further complicate this process and make delivering patient-centered care, even more challenging [22, 25].

**ii. Other prescribers:** Multimorbid patients tend to visit a greater number of physicians, which could include specialists. This can have several effects on the patients’ care. It has been shown that those patients who visit four or more physicians experience problems such as conflicting medical advice, lack of access to laboratory test results and more commonly, duplication of laboratory tests from multiple prescribers [26].

It was apparent in our study, that GPs’ prescribing is influenced by specialist recommendations. One GP disclosed, they considered which specialist initiated the medicine, before changing the prescription.

“So if they’ve seen a cardiologist and were put on a statin, you feel very nervous about stopping it for example. There’s no doubt that you know a specialist assessment, commenced on a specific drug, you know it does make you reluctant to change things, yeah, it does have that, it influences you.” GP7

In addition, GPs believed that patients have the perception that physicians in hospitals, or specialists in private clinics have more knowledge than GPs practising in primary health care. This could disrupt the trust in patient-GP relationships, leading to compromised shared-decision making between the patient and the GP.

“Yeah, look the big doctor in the white coat in the big house on the hill always knows more than the GP especially the house surgeon who might have a brief amount of experience and does what they’re told and one of the issues with this process is, experienced GP’s still think that the doctor up the road knows more.” GP9

Specialists who operate on a single disease paradigm without an overview of the ‘whole patient’ can lead to fragmented care. Single-disease care is antagonistic to the goals of GPs in primary care. GPs receive poor communication from other care providers in multimorbidity [22]. This uncertainty makes it more difficult for GPs to make prescribing decisions with confidence and impedes GPs from delivering patient-centred care to their patients [22].

**iii. Residential care staff:** Nurses have a significant role in caring for people living in residential care. Most of the information GPs collect is sourced from nurses instead of a direct conversation with the patient. As a result, biased opinions could be communicated and shared-decision making between the patient and the GP could be compromised.

“We are relying on the nursing staff to feedback information to us, and often that’s not brilliant, hence we’ve lost that kind of one to one with the patient, which makes it much harder to decide how to prescribe, because you’re being biased by other people.” GP3

In addition, nurses could encourage GPs to prescribe certain medicines; in particular those medicines with a sedative effect.

“Uh, some nurses do push for medicines to be prescribed.” GP8

“I do think sometimes you wonder who are we treating? Are we treating the nursing staff who can’t face somebody calling out at night, or are we treating (an) actual patient who may be very well, happen to be calling out once or twice a night at one o’clock, but then fall back to sleep, you know?” GP9

### C. Policy and processes

**i. Communication at transfer points of healthcare:** Transfer points of healthcare include admission into hospital from a patient’s home or a residential care facility and discharge from the hospital. At these points, older patients are particularly vulnerable as they find it more difficult to adapt or adjust to different environments.

“I recently had a patient who went into hospital, and the hospital stopped stuff, which is great. Then they came back into the rest home, a different environment, and they were upset. So this is the battle, do you start it again? Because older people, patients, don’t do well from one place to another place. I think they would rather just always be in the same place.” GP9

Fragmented patient care may result at these points of transfer, consequent to multiple physicians providing medical care and prescribing. GPs in this study highlighted that discharge summaries are usually written clearly, however they felt that they often lack detail around how long the new medicine should be continued for. This contributes to GPs continuing the prescription of medicines for longer than initially intended.

“Discharge summaries are clear, but don’t necessarily mention whether medicines should be continued long-term.” GP1

Several GPs in this study spoke about the lack of communication between hospital physicians and GPs in primary care. The lack of communication led to patients being prescribed medicine regimens consistent with hospital formulary or guidelines, but potentially inappropriate to a multimorbid older patient.

“Some recognition of the problem from secondary care is important, because if people are admitted to hospital, they tend to be put on standard regimes without taking light of their medication and they tend to be looked at in a silos. If they have an MI (myocardial infarction), they’d come out with 40mg of simvastatin and the other side of things won’t be looked at.” GP1

“The challenges are stopping hospital doctors giving unnecessary medicines. Hospitals are focussed on the single issues. They tend to focus on single issues and add, add, and add medications. I think there also tends to be a “just keep going” because it’s been started.” GP5

Another previously documented challenge related to transitions in healthcare, is when the primary prescriber changes, and the knowledge of the rationale or indication for the medication is lost at the transfer. In this case the responsibility for stopping inappropriate medicines is devolved to the new prescriber, but not the necessary information required for decision making [27].

**ii. Time and funding constraints:** The majority of GPs in this study visited residents at the residential care facility at unscheduled times. This meant patient care was fitted around other responsibilities, such as prioritizing time for their own patients in the medical practice.

“You rush at lunchtime or you rush before work, so you’re often fitting it (rest home prescribing) around other things.” GP2

“As a goal, just to reduce the number of tablets for the sake of it. No, I don’t do that at all. Maybe I should, but life’s busy.” GP4

GPs felt that time constraints may have stopped them addressing all of the patient’s concerns, and led to suboptimal medicine management.

“Time constraints, I’ve got 15 minutes, she’s worried about ‘x, y, & z’. I’m really concerned about this one, so we’ve got all that negotiation, and I’m not about to sit down and go through and spend 20 minutes or half an hour, because I’ve got the waiting room full. Those are real drivers.” GP4

Similar limitations were echoed in other studies. In a qualitative study conducted by Luijks *et al*., Dutch GPs revealed that there was insufficient time and compensation for consistently putting their main objectives into practice [24]. Fried *et al*. discussed that current reimbursement systems, fail to acknowledge the complexities of caring for older people with multiple conditions [23].

**iii. Nursing home policies and a chaotic prescribing environment:** GPs reported their frustrations regarding lack of clarity and consistency of clinical documentation among residential care facilities. Lack of decision-support systems in residential care challenged GPs. They found that routine paper work was burdensome, inefficient and increased the scope for error. Transmitting a hard copy of medicine charts via fax between the residential care facility, the pharmacy and the medical practice compromised the quality and legibility of the medicine charts leading to avoidable medicine and administration errors. As a result, the prescribing environment for GPs in residential care, can sometimes be chaotic. These issues may diminish in the future, as residential aged care facilities in New Zealand adopt electronic prescribing systems[28].

“You can’t get consistency, you’ve got private rest homes, and they’ve all got different systems. For instance; for three-monthly reviews: you go and see a patient; you look at the drug chart and examine the patient. You write something in the notes, and then they want another bit of paper to be filled out that says you’ve done a review on the medicines apparently.” GP3

“None of the local rest homes are set up for computerised records which drives me nuts. We are fully computerised (in) general practice these days and when we come to rest homes we go back to this antiquated system of having to handwrite things. Especially, I hate, hate, hate handwriting prescriptions because the room for error goes up exponentially, and then with the multiple faxing of charts.” GP5

### Theme 4: Deprescribing enablers

GPs in this study recognised the need for support when prescribing for multimorbid patients. Several possible deprescribing enablers came to light, which can serve as a platform for improving medicine management in older people.

#### 1) Pharmacist medicine reviews in a multidisciplinary integrated approach

In this study, time constraints were identified as a limitation preventing physicians from reviewing medicine lists for older people. This impedes the process of deprescribing medicines noted to be unnecessary and/or harmful. One way to implement deprescribing is to conduct comprehensive deprescribing reviews. Physicians and pharmacists have worked together using a multidisciplinary approach to reduce polypharmacy and inappropriate medication use (IMU) in older people by carrying out comprehensive medicine reviews [13–16].

The GPs in this study, perceived that treating multimorbid patients, required a collective effort from different health care professional groups. This approach could really help deliver patient-centred care, especially if the patient and/or their family are involved in the process.

“I think if you made medicine reviews compulsory, I think it could be very useful. When people have incentives, for example, if they had to do it for accreditation, they will do it, otherwise it’s not going to happen. It’s simple, you know? People just don’t have, I mean we’re all busy, and don’t have tons of time. I think we need to carry out medication reviews, and not miss people out. Sometimes its good to have somebody else look at it, so working together with a pharmacist is a good idea. Because I think two pairs of eyes looking at the same page, often gets better results than one person looking at a patient.” GP9

“Its little things you know, such as, how about considering a medicine review on this patient as they meet the criteria you know for a medicine review?” GP7

“I’m not against talking with a pharmacist or a geriatrician for half a dozen, ten patients that you look through and even a consultant, and say we’re looking at that for that person. I mean that to me (speaking to a pharmacist and a geriatrician about some patients) would be a reasonable thing, as well (if) it didn’t take too much time and it wasn’t too difficult to prepare. So you could make it more certain with ideas from a pharmacist and a consultant about that. And I would see that as worth thinking about.” GP10

“Well the help of a clinical pharmacist, or certainly a very good nurse manager to help, or the cardiac nurse specialist, because if we’re decreasing medication we’ve got to keep an eye on heart failure, blood pressure, blood sugars (glycated haemoglobin) kind of thing. But having a clinical pharmacist involved would be great. I mean ideally a physician, a clinical pharmacist and the GP, and the nurse would be great. You know, the whole mixture” GP2

Several GPs embraced the idea of pharmacists’ involvement in a multidisciplinary team to review medicines, but had concerns about the delivery of the service. The primary concern was that pharmacists may offer recommendations that may have already been recognised by the GP and these may have not been implemented for compelling clinical reasons. These reasons are often not communicated to or recognised by the pharmacists. GPs were positive about the potential for inter-professional partnerships. In a residential care setting, pharmacists have access to residents’ clinical information and are well placed to make recommendations for GPs to consider. In contrast pharmacists working in the community do not have access to patient information. As a result, GPs fear that the pharmacists’ recommendations might not be relevant in this setting. In addition, lack of resources hinders these multidisciplinary meetings from occurring regularly in practice.

“I think it’s good to know the pharmacists if you can, but it doesn’t have to be a local pharmacist. It’s just that locality means you build relationship with people more easily.” GP6

“The pharmacist down the road are sort of part of the team, but they’re in a different building, and we never see them. They’re a voice on the phone, they do rollover and they lack information; there’s no doubt about that. They don’t know what Mrs. Bloggs has got or why we’re treating them for half the stuff. We would be better if they had the time to, for the pharmacists to sit down with the physician and say you know, it’s possible that you could stop ‘x’ and ‘y’. But how can they do that if they don’t know what the patient’s got? I guess, if you’re part of the treating team, you’re part of the treating team. And that’s the critical thing. And if you’re not, you’re not.” GP4

#### 2) Adequate reimbursement

GPs in this study felt that the overall structure of residential care prescribing and reimbursement systems are disorganised. Prescribing for older people in residential care is viewed as cumbersome for many, as time constraints and limited resources prevent this process from being carried out efficiently. In addition, they felt that they received insufficient compensation for the amount of work and attention that this area of prescribing requires. These opinions were not exclusive to our study and are similar to the views of GPs in other studies [12, 19, 24].

“The challenges are around time coordination, and um, GPs invariably complain they don’t have enough time. But being cynical, not much here, and long enough in the tooth, that if you pay people adequately, they will make the time. A lot of this is under-funded, and it takes time outside people’s main surgery working hours. GP5

“Your first challenge is; you go to the rest home. You try and find a nurse. You can never find one. You try and find the notes, hard to find. You can’t find the medicine chart, it could be on the rounds somewhere. It’s not computerised, it doesn’t link with our technical notes at the medical practice, so quality just goes down. It shouldn’t be, but at the practice we’ve got the computer, we’ve got light, we don’t have a darkened room in a rest home, and we can actually see what’s going on.” GP3

If I don’t have my rest home rounds by 7:30 in the morning at least 4 days a week, I barely finish up with the rest of my rest home work then occurring jammed in at lunchtime or at 6 o’clock at night, 7 o’clock at night, when you… the nurse may no longer be on duty, the patient’s tired, it all turns to custard. So it is not accorded a high, a very high priority and does take place at these extreme ends of the day, and whereas if it’s adequately funded, for you know, 8–8:30 in the morning, and it’s properly funded, and everybody’s there and ready to go, GP’s will go. But while it’s as ad-hoc, as it is, it’s seen as a chore and it’s not well done.” GP5

“Um, I think having adequate funding for older people so that you have time to really think about what they are having so, and Care Plus (a recent government funding initiative) has been good from that point of view, because it gives some extra funding for people like this who have complex health problems. So that has been a real help having enough you know, that recognition of the time involved in reviewing people adequately in rest homes. So that’s really, so that’s’ fine at the moment. I hope they don’t remove that, because it just puts the cost on patients if you’re going to give time to reviewing their medication. So you know there’s nothing additional that I would do, yeah.” GP6

#### 3) Better communication between physicians at health interfaces

Clear and transparent communication is essential between GPs in primary care, specialists and hospital physicians. Integrated health care meetings between GPs and specialists, and information technology to clearly outline patient plans in terms of prognosis and care, are some strategies that can improve seamless care for a multimorbid older individual. Bidirectional communication between GPs in primary care and physicians at the hospital is crucial. This would improve the suitability of medicines prescribed to patients and in some cases, may prevent inappropriate medicines being prescribed. Furthermore, better communication would help physicians to ‘speak with one voice’ which would result in greater satisfaction for both physicians and patients, as different stories provoke distrust [12, 22].

“The reverse of hospitals putting them on medications, is that hospitals stop medications sometimes, and a week later they start them on all of the medicines again, so that sort of communication needs to be taken into consideration.” GP1

“If we could stop hospital physicians prescribing single issue medicines for compromised older people, we’d reduce our problems by 50% overnight.” GP5

“I think we are not daring enough, and I think the communication between the hospital and us (GPs in primary care) could be far better.” GP9

#### 4) Deprescribing guidelines, GP education and GP empowerment

Experiences of practising physicians in this study and other studies, suggest that they struggle with the uncertainties of applying disease-specific guidelines to older people with multimorbidity [23]. One qualitative study, by Smith *et al*., reported that having a multimorbidity focus in GP training is important as GPs expressed that they lacked confidence and felt that they needed more training and clinical support [25]. Another study by Herzog et *al*., reported that specific geriatric training for GPs is likely to have a positive effect and may help overcome some of the barriers outlined earlier [19]. In the present study, one GP also highlighted the need for improving GPs’ awareness on such issues.

“Improve GPs’ awareness or education on the issues (involved with prescribing for multimorbid patients), and um, perhaps the medicine review service.” GP7

Improving GPs’ awareness can be augmented by positive testimonies from GPs. This may also help empower GPs, and could encourage them to deprescribe. One GP in this study spoke about the positive outcomes they observed after implementing deprescribing.

“Since I’ve started to look at that more globally, the number of medicines I’m prescribing on average for patients in rest homes is about 50% of what I was prescribing a year ago and they aren’t falling off their perch in greater numbers. Patients like it (being prescribed less medicines). They say oh, a whole big meal of pills, and you know, people are generally better. People wake up, they’re less nauseated, they have fewer falls, all those sorts of things, yeah. GP5

In addition, this study illustrates that GPs feel pressured into continuing the prescription of certain medicines initiated by specialists; even if they question the medicines’ suitability. GPs also find it more difficult to convince their patients of certain changes, as people may perceive physicians in the hospital to have more experience or knowledge.

“Yeah, look the big doctor in the white coat in the big house on the hill always knows more than the GP especially the house surgeon who might have a brief amount of experience and does what they’re told and one of the issues with this process is, experienced GP’s still think that the doctor up the road knows more.” GP9

Therefore, it is important to ensure that current GP training meets the needs of GPs, and is focused around delivering patient-centered care for older people with multiple comorbidities. In addition, further work is required to create and disseminate clinical tools, suitable for use in primary care practice as GPs need different approaches, to help them deliver medical care that fulfills their patients’ priorities [23]. These strategies need to allow GPs to employ flexibility in implementing prescribing guidelines, while responding to the individuals’ needs and preferences [29]. This will in turn empower GPs and provide them with the confidence needed to be experts in prioritising medicines and stopping medicines initiated by specialists who would have dealt with one particular patient issue, without considering all other aspects of the patient’s care.

## Strengths and Limitations

A qualitative semi-structured design enabled us to capture the various challenges and opinions GPs faced with deprescribing. The qualitative methods used were appropriate for exploring the topic of deprescribing challenges and enablers, and they met the criteria for “trustworthiness” established by Lincoln and Guba in 1986 [30, 31]. Credibility was achieved through prolonged engagement with the data, reading and examining in-depth interviews and interview notes written by NA. Transcriptions were read twice, checked and coded initially by NA. Coding was independently checked by JT. Transferability was achieved by independent checking of interpretations made in this article, by JT, PN and DM.

Implementation of the Theoretical Domains Framework [17], elicited both environmental and physician-related factors involved in implementing deprescribing; a practice which is currently not widespread. The Theoretical Domains Framework covered a wide range of topics, and was easy to implement during the analysis of the interviews.

Due to the limited participation of GPs, our respondent sample is not representative of all GPs, regions or jurisdictions. GPs were aware of the interviewer being a pharmacist hence their opinions on involving pharmacists could have been skewed. Their opinions might also have influenced positively or negatively by their experiences of working alongside pharmacists compared with physicians in other jurisdictions who have not worked with pharmacists. GPs might have provided a socially desirable response to some interview questions. For example, when asked if deprescribing guidelines would be useful in their everyday clinical practice, GPs may have expressed the need for them. However, in their everyday practice, they might not like to refer to them.

Interviews were conducted face-to-face rather than utilising a focus group design. The advantages of such interviews may include that GPs would be more comfortable expressing their honest and unbiased opinions in this setting, rather than in a focus group, where peer pressure might play a role in influencing their responses. Disadvantages of conducting interviews could include having limited discussion and expansion of the reasoning behind the different responses. A focus group could have offered a greater deal of diversity and an opportunity to discuss different issues. However, a focus group would have been more difficult to conduct in an impartial manner to ensure that every participant’s opinions were heard and noted.

This study is exploratory in nature with the sole purpose of understanding barriers and enablers to deprescribing in a residential care setting. The main limitation is the sample size, however saturation of ideas was reached within the sample. Despite some limitations, this study clearly highlights the challenges of deprescribing in the residential care setting. It also brought to light, based on the GPs’ cumulative opinions and experiences of prescribing for older people in this setting, possible enablers to deprescribing.

## Conclusion

The process of deprescribing in a multimorbid older individual is laced with many challenges for GPs. These include time and process pressures, a chaotic prescribing environment, a lack of value assigned to deprescribing in medical care processes and in families’ perceptions and a lack of specific evidence-based guidelines applicable for the cessation of medicines in older people. Most GPs also felt cautious when deprescribing, because they feared causing disease relapse or drug withdrawal symptoms in patients. They also feared individuals’ misperception of patients’ coincident deterioration. Social pressures such as feedback from nurses and a sense of taking on board specialist recommendations influenced GPs’ prescribing. Process barriers involved poor communication between physicians at different health care interfaces to deliver patient-centered care. Enablers of deprescribing included adequate imbursement, improved communication between physicians at health interfaces and appropriate deprescribing guidelines. Involvement of pharmacists in multidisciplinary teams was perceived to be potentially beneficial. The results of this qualitative study invite further development and testing of deprescribing guidance for GPs to follow in a residential care setting. Future research should be directed at investigating the benefits and risks of deprescribing as well as possible tools and changes to policies and processes to address barriers and enablers, and provide support for patient-centred care delivered in a residential care setting.
